# Supplementary material for: A Comprehensive Review for the Surveillance of Human Pathogenic Microorganisms in Shellfish
Source: Microorganisms. 2023 Aug 31;11(9):2218. doi: 10.3390/microorganisms11092218 (PMC10537662; doi:10.3390/microorganisms11092218)
Supplement: Supplementary file 1 [file microorganisms-11-02218-s001.zip › microorganisms-2569307-supplementary.pdf]

Supplemental Table S1. List of countries where human enteric bacteria were detected in marine or freshwater BMS, with references. This table does not include marine bacteria detection (such as *Vibrio* or *aeromonas*)

Country	*Escherichia*	*Salmonella*	*Shigella*	*Klebsiella*	*Campylobacter*	*Arcobacter*	*Helocibacter*	*Clostridium*	*Enterococcus*	Reference
Argentina										
Australia					+					[1]
Brazil	+									[2]
		+	-							[3]
									+	[4]
		+								[5]
Canada		+								[6]
China										
Croatia					+					[7]
Costa Rica	+									[8]
Denmark										
Egypt								+		[9]
	+									[10]
Finland										
France					+					[11]
	+	+			+				+	[12]
					+					[1]
	+									[13]
	+			+						[14]
		+								[15]
Greece	+	-								[16]
India						+				[17]
Ireland									+	[18]
	+	+			+			+		[19]
Italy								+		[20]
								+		[21]

				+						[22]
						+				[23]
Japan								+		[24]
		+	-							[25]
			+							[26]
Netherlands (the)					+					[27]
Norway	+			+						[28]
				+						[29]
Portugal	+			+						[30]
Spain								+		[31]
						+				[32]
							+			[33]
		+								[34]
Thailand					+					[35]
		+								[36]
	+	+	+							[37]
	+									[38]
Tunisia	+	-								[39]
	+			+						[40]
United Kingdom		+			+					[19]
United States of America					+					[41]
								+		[42]
	+								+	[43]
		+								[44]

+ : bacteria detected in BMS

- : bacteria searched but not detected

References

1. Gourmelon, M.; Boukerb, A.M.; Nabi, N.; Banerji, S.; Joensen, K.G.; Serghine, J.; Cormier, A.; Megraud, F.; Lehours, P.; Alter, T.; et al. Genomic Diversity of *Campylobacter Lari* Group Isolates from Europe and Australia in a One Health Context. *Appl. Environ. Microbiol.* **2022**, *88*, e0136822, doi:10.1128/aem.01368-22.

2. Vásquez-García, A.; Oliveira, A.P.S.C. de; Mejia-Ballesteros, J.E.; Godoy, S.H.S. de; Barbieri, E.; Sousa, R.L.M. de; Fernandes, A.M. *Escherichia Coli* Detection and Identification in Shellfish from Southeastern Brazil. *Aquaculture* **2019**, *504*, 158–163, doi:10.1016/j.aquaculture.2019.01.062.
3. Ristori, C.A.; Iaria, S.T.; Gelli, D.S.; Rivera, I.N.G. Pathogenic Bacteria Associated with Oysters (Crassostrea Brasiliana) and Estuarine Water along the South Coast of Brazil. *Int. J. Environ. Health Res.* **2007**, *17*, 259–269, doi:10.1080/09603120701372169.
4. Sacramento, A.G.; Fernandes, M.R.; Sellera, F.P.; Dolabella, S.S.; Zanella, R.C.; Cerdeira, L.; Lincopan, N. VanA-Type Vancomycin-Resistant *Enterococcus Faecium* ST1336 Isolated from Mussels in an Anthropogenically Impacted Ecosystem. *Mar. Pollut. Bull.* **2019**, *142*, 533–536, doi:10.1016/j.marpolbul.2019.04.014.
5. de Souza, R.V.; Moresco, V.; Miotto, M.; Souza, D.S.M.; de Campos, C.J.A. Prevalence, Distribution and Environmental Effects on Faecal Indicator Bacteria and Pathogens of Concern in Commercial Shellfish Production Areas in a Subtropical Region of a Developing Country (Santa Catarina, Brazil). *Environ. Monit. Assess.* **2022**, *194*, 286, doi:10.1007/s10661-022-09950-5.
6. Tamber, S.; Montgomery, A.; Eloranta, K.; Buenaventura, E. Enumeration and Survival of *Salmonella Enterica* in Live Oyster Shellstock Harvested from Canadian Waters. *J. Food Prot.* **2020**, *83*, 6–12, doi:10.4315/0362-028X.JFP-19-318.
7. Jurinović, L.; Ječmenica, B.; Džafić, N.; Brlek Gorski, D.; Šimpraga, B.; Krstulović, F.; Amšel Zelenika, T.; Humski, A. First Data on *Campylobacter* Spp. Presence in Shellfish in Croatia. *Pathogens* **2022**, *11*, 943, doi:10.3390/pathogens11080943.
8. Symonds, E.M.; Young, S.; Verbyla, M.E.; McQuaig-Ulrich, S.M.; Ross, E.; Jiménez, J.A.; Harwood, V.J.; Breitbart, M. Microbial Source Tracking in Shellfish Harvesting Waters in the Gulf of Nicoya, Costa Rica. *Water Res.* **2017**, *111*, 177–184, doi:10.1016/j.watres.2017.01.004.
9. Ezzeldeen, N.; Ammar, A.; shalaby, B.; Elhariri, M.; Omar, W. Rapid Detection of *Clostridium Perfringens* in Seafood. *Adv. Environ. Biol.* **2016**, *10*, 174–181.
10. Al Qabili, D.M.A.; Aboueisha, A.-K.M.; Ibrahim, G.A.; Youssef, A.I.; El-Mahallawy, H.S. Virulence and Antimicrobial-Resistance of Shiga Toxin-Producing *E. Coli* (STEC) Isolated from Edible Shellfish and Its Public Health Significance. *Arch. Microbiol.* **2022**, *204*, 510, doi:10.1007/s00203-022-03114-2.
11. Boukerb, A.M.; Penny, C.; Serghine, J.; Walczak, C.; Cauchie, H.-M.; Miller, W.G.; Losch, S.; Ragimbeau, C.; Mossong, J.; Mégraud, F.; et al. *Campylobacter Armoricus* Sp. Nov., a Novel Member of the *Campylobacter Lari* Group Isolated from Surface Water and Stools from Humans with Enteric Infection. *Int. J. Syst. Evol. Microbiol.* **2019**, *69*, 3969–3979, doi:10.1099/ijsem.0.003836.
12. Rincé, A.; Balière, C.; Hervio-Heath, D.; Cozien, J.; Lozach, S.; Parnaudeau, S.; Le Guyader, F.S.; Le Hello, S.; Giard, J.-C.; Sauvageot, N.; et al. Occurrence of Bacterial Pathogens and Human Noroviruses in Shellfish-Harvesting Areas and Their Catchments in France. *Front. Microbiol.* **2018**, *9*, 2443, doi:10.3389/fmicb.2018.02443.
13. Balière, C.; Rincé, A.; Blanco, J.; Dahbi, G.; Harel, J.; Vogeleer, P.; Giard, J.-C.; Mariani-Kurkdjian, P.; Gourmelon, M. Prevalence and Characterization of Shiga Toxin-Producing and Enteropathogenic *Escherichia Coli* in Shellfish-Harvesting Areas and Their Watersheds. *Front. Microbiol.* **2015**, *6*.
14. Delannoy, S.; Hoffer, C.; Youf, R.; Dauvergne, E.; Webb, H.E.; Brauge, T.; Tran, M.-L.; Midelet, G.; Granier, S.A.; Haenni, M.; et al. High Throughput Screening of Antimicrobial Resistance Genes in Gram-Negative Seafood Bacteria. *Microorganisms* **2022**, *10*, 1225, doi:10.3390/microorganisms10061225.
15. Gourmelon, M.; Solen, L.; Pascal, G.; Clémence, B.; Nicolas, S. Prevalence and Characterization of *Salmonella* Spp. in Three Shellfish-Harvesting Areas in France.
16. Lattos, A.; Chaligiannis, I.; Papadopoulos, D.; Giantsis, I.A.; Petridou, E.I.; Vafeas, G.; Staikou, A.; Michaelidis, B. How Safe to Eat Are Raw Bivalves? Host Pathogenic and Public Health Concern

Microbes within Mussels, Oysters, and Clams in Greek Markets. *Foods Basel Switz.* **2021**, *10*, 2793, doi:10.3390/foods10112793.

17. Laishram, M.; Rathlavath, S.; Lekshmi, M.; Kumar, S.; Nayak, B.B. Isolation and Characterization of *Arcobacter* Spp. from Fresh Seafood and the Aquatic Environment. *Int. J. Food Microbiol.* **2016**, *232*, 87–89, doi:10.1016/j.ijfoodmicro.2016.05.018.
18. Wilson, I.G.; McAfee, G.G. Vancomycin-Resistant *Enterococci* in Shellfish, Unchlorinated Waters, and Chicken. *Int. J. Food Microbiol.* **2002**, *79*, 143–151, doi:10.1016/s0168-1605(02)00063-6.
19. Wilson, I.G.; Moore, J.E. Presence of *Salmonella* Spp. and *Campylobacter* Spp. in Shellfish. *Epidemiol. Infect.* **1996**, *116*, 147–153.
20. Pasquale, V.; Romano, V.J.; Rupnik, M.; Dumontet, S.; Cižnár, I.; Aliberti, F.; Mauri, F.; Saggiomo, V.; Krovacek, K. Isolation and Characterization of *Clostridium Difficile* from Shellfish and Marine Environments. *Folia Microbiol. (Praha)* **2011**, *56*, 431–437, doi:10.1007/s12223-011-0068-3.
21. Agnoletti, F.; Arcangeli, G.; Barbanti, F.; Barco, L.; Brunetta, R.; Cocchi, M.; Conedera, G.; D'Este, L.; Drigo, I.; Spigaglia, P.; et al. Survey, Characterization and Antimicrobial Susceptibility of *Clostridium Difficile* from Marine Bivalve Shellfish of North Adriatic Sea. *Int. J. Food Microbiol.* **2019**, *298*, 74–80, doi:10.1016/j.ijfoodmicro.2019.03.003.
22. Simoni, S.; Leoni, F.; Veschetti, L.; Malerba, G.; Carelli, M.; Lleò, M.M.; Brenciani, A.; Morroni, G.; Giovanetti, E.; Rocchegiani, E.; et al. The Emerging Nosocomial Pathogen *Klebsiella Michiganensis*: Genetic Analysis of a KPC-3 Producing Strain Isolated from Venus Clam. *Microbiol. Spectr.* **2022**, *11*, e04235-22, doi:10.1128/spectrum.04235-22.
23. Mottola, A.; Bonerba, E.; Figueras, M.J.; Pérez-Cataluña, A.; Marchetti, P.; Serraino, A.; Bozzo, G.; Terio, V.; Tantillo, G.; Di Pinto, A. Occurrence of Potentially Pathogenic *Arcobacters* in Shellfish. *Food Microbiol.* **2016**, *57*, 23–27, doi:10.1016/j.fm.2015.12.010.
24. Yanagimoto, K.; Uematsu, K.; Yamagami, T.; Haramoto, E. The Circulation of Type F *Clostridium Perfringens* among Humans, Sewage, and Ruditapes Philippinarum (Asari Clams). *Pathog. Basel Switz.* **2020**, *9*, 669, doi:10.3390/pathogens9080669.
25. Hara-Kudo, Y.; Konuma, H.; Kamata, Y.; Miyahara, M.; Takatori, K.; Onoue, Y.; Sugita-Konishi, Y.; Ohnishi, T. Prevalence of the Main Food-Borne Pathogens in Retail Food under the National Food Surveillance System in Japan. *Food Addit. Contam. Part Chem. Anal. Control Expo. Risk Assess.* **2013**, *30*, 1450–1458, doi:10.1080/19440049.2012.745097.
26. Terajima, J.; Tamura, K.; Hirose, K.; Izumiya, H.; Miyahara, M.; Konuma, H.; Watanabe, H. A Multi-Prefectural Outbreak of *Shigella Sonnei* Infections Associated with Eating Oysters in Japan. *Microbiol. Immunol.* **2004**, *48*, 49–52, doi:10.1111/j.1348-0421.2004.tb03486.x.
27. Teunis, P.; Havelaar, A.; Vliegenthart, J.; Roessink, G. Risk Assessment of *Campylobacter* Species in Shellfish: Identifying the Unknown. *Water Sci. Technol.* **1997**, *35*, 29–34, doi:10.1016/S0273-1223(97)00230-8.
28. Grevskott, D.H.; Svanevik, C.S.; Sunde, M.; Wester, A.L.; Lunestad, B.T. Marine Bivalve Mollusks As Possible Indicators of Multidrug-Resistant *Escherichia Col*i and Other Species of the *Enterobacteriaceae* Family. *Front. Microbiol.* **2017**, *8*, 24, doi:10.3389/fmicb.2017.00024.
29. Håkonsholm, F.; Hetland, M.A.K.; Svanevik, C.S.; Sundsfjord, A.; Lunestad, B.T.; Marathe, N.P. Antibiotic Sensitivity Screening of *Klebsiella* Spp. and *Raoultella* Spp. Isolated from Marine Bivalve Molluscs Reveal Presence of CTX-M-Producing *K. Pneumoniae*. *Microorganisms* **2020**, *8*, 1909, doi:10.3390/microorganisms8121909.
30. Freire, S.; Grilo, T.; Rodrigues, B.; Oliveira, R.; Esteves, C.; Marques, A.; Poirel, L.; Aires-de-Sousa, M. ESBL- and Carbapenemase-Producing *Escherichia Coli* and *Klebsiella Pneumoniae* among Bivalves from Portuguese Shellfish Production Areas. *Microorganisms* **2023**, *11*, 415, doi:10.3390/microorganisms11020415.
31. Candel-Pérez, C.; Zapata-Galián, E.; López-Nicolás, R.; Ros-Berruezo, G.; Martínez-Graciá, C. Presence of Toxigenic *Clostridioides (Clostridium) Difficile* in Edible Bivalve Mollusks in Spain. *Food Sci. Technol. Int. Cienc. Tecnol. Los Aliment. Int.* **2020**, *26*, 413–419, doi:10.1177/1082013219894092.

32. Morejón, I.F.B.; González, A.; Ferrús, M.A. Detection, Identification, and Antimicrobial Susceptibility of *Arcobacte*r Spp. Isolated from Shellfish in Spain. *Foodborne Pathog. Dis.* **2017**, *14*, 238–243, doi:10.1089/fpd.2016.2202.
33. Pina-Pérez, M.C.; González, A.; Moreno, Y.; Ferrús, M.A. *Helicobacter Pylori* Detection in Shellfish: A Real-Time Quantitative Polymerase Chain Reaction Approach. *Foodborne Pathog. Dis.* **2019**, *16*, 137–143, doi:10.1089/fpd.2018.2495.
34. Martinez-Urtaza, J.; Saco, M.; Hernandez-Cordova, G.; Lozano, A.; Garcia-Martin, O.; Espinosa, J. Identification of *Salmonella* Serovars Isolated from Live Molluscan Shellfish and Their Significance in the Marine Environment. *J. Food Prot.* **2003**, *66*, 226–232, doi:10.4315/0362-028X-66.2.226.
35. Soonthornchaikul, N.; Garelick, H. Antimicrobial Resistance of *Campylobacter* Species Isolated from Edible Bivalve Molluscs Purchased from Bangkok Markets, Thailand. *Foodborne Pathog. Dis.* **2009**, *6*, 947–951, doi:10.1089/fpd.2008.0236.
36. Atwill, E.R.; Jeamsripong, S. Bacterial Diversity and Potential Risk Factors Associated with *Salmonella* Contamination of Seafood Products Sold in Retail Markets in Bangkok, Thailand. *PeerJ* **2021**, *9*, e12694, doi:10.7717/peerj.12694.
37. Jeamsripong, S.; Chuanchuen, R.; Atwill, E.R. Assessment of Bacterial Accumulation and Environmental Factors in Sentinel Oysters and Estuarine Water Quality from the Phang Nga Estuary Area in Thailand. *Int. J. Environ. Res. Public. Health* **2018**, *15*, 1970, doi:10.3390/ijerph15091970.
38. Jeamsripong, S.; Atwill, E.R. Modelling of Indicator *Escherichia Coli* Contamination in Sentinel Oysters and Estuarine Water. *Int. J. Environ. Res. Public. Health* **2019**, *16*, 1971, doi:10.3390/ijerph16111971.
39. Gdoura, M.; Sellami, H.; Khannous, L.; Ketata, N.; Neila, I.B.; Traore, A.I.; Chekir, Z.; Gdoura, R. Elimination of *Escherichia Coli* and *Salmonella* in Clam by Using Zeolite in a Station of Depuration. *Water Environ. Res.* **2017**, *89*, 856–861.
40. Mani, Y.; Mansour, W.; Lupo, A.; Saras, E.; Bouallègue, O.; Madec, J.-Y.; Haenni, M. Spread of BlaCTX-M-15-Producing *Enterobacteriaceae* and OXA-23-Producing *Acinetobacter Baumanni*i Sequence Type 2 in Tunisian Seafood. *Antimicrob. Agents Chemother.* **2018**, *62*, e00727-18, doi:10.1128/AAC.00727-18.
41. Abeyta, C.; Deeter, F.G.; Kaysner, C.A.; Stott, R.F.; Wekell, M.M. *Campylobacter Jejuni* in a Washington State Shellfish Growing Bed Associated With Illness. *J. Food Prot.* **1993**, *56*, 323–325, doi:10.4315/0362-028X-56.4.323.
42. Norman, K.N.; Harvey, R.B.; Andrews, K.; Hume, M.E.; Callaway, T.R.; Anderson, R.C.; Nisbet, D.J. Survey of *Clostridium Difficile* in Retail Seafood in College Station, Texas. *Food Addit. Contam. Part Chem. Anal. Control Expo. Risk Assess.* **2014**, *31*, 1127–1129, doi:10.1080/19440049.2014.888785.
43. Korajkic, A.; Badgley, B.D.; Brownell, M.J.; Harwood, V.J. Application of Microbial Source Tracking Methods in a Gulf of Mexico Field Setting. *J. Appl. Microbiol.* **2009**, *107*, 1518–1527, doi:10.1111/j.1365-2672.2009.04351.x.
44. Brands, D.A.; Inman, A.E.; Gerba, C.P.; Maré, C.J.; Billington, S.J.; Saif, L.A.; Levine, J.F.; Joens, L.A. Prevalence of *Salmonella* Spp. in Oysters in the United States. *Appl. Environ. Microbiol.* **2005**, *71*, 893–897, doi:10.1128/AEM.71.2.893-897.2005.

Supplemental Table S2. List of countries where human viruses were detected in marine or freshwater BMS, with references.

Country	AdV	AiV	AsV	EV	HAV	HEV	NoV	RV	SaV	SARS-CoV-2	Reference
Argentina							+	+			[1]
Australia					-		-				[2]
							+				[3]
Brazil			-				+				[4]
	+			+			+				[5]
					+		+	+			[6]
	+						+	+			[7]
	+						+	+			[8]
Bulgaria							+				[9]
Cameroon					+		+				[10]
Canada							+				[11]
China							+	+			[12]
						+					[13]
Croatia								+		-	[14]
Denmark						-		-			[15]
Finland*					-	-	+				[16]
France		+	+	+	-		+	+			[17]
				+	+		+	+			[18]
		-		+	-	-	+	+	+		[19]
						-					[20]
							+			-	[21]
Greece	+			+	+		+				[22]
India	+			+	-		-				[23]
							+				[24]
Ireland							+				[25]
Italy	+	+	+		-	-	+	+	+		[26]
	+	+	+		+	-	+	+	+		[27]
	+			+	+	+	+	-			[28]

		+									[29]
	+		-	+	-	-	+				[30]
										+	[31]
Japan	+	+	-		+		+	+			[32]
									+		[33]
										-	[34]
South Korea							+				[35]
	+			+							[36]
Mexico					+		+				[37]
Montenegro							+				[38]
Morocco	+										[39]
		+		+	+		+		+		[40]
						+					[41]
Netherlands (the)			-	+	-		-	-			[42]
					+		+				[43]
New Zeland	+						+				[44]
Norway	+						+				[45]
							+		+		[46]
Poland					+		+				[47]
Portugal				+	+		+				[48]
						-	+				[49]
Singapore					+	+	+	-			[50]
Slovenia							+				[51]
Sweden	+			+	-		+				[22]
Taiwan	+						+				[52]
Thailand					+	-					[53]
								+			[54]
							+				[55]
Tunisia		+									[56]
			+	+	+		+				[57]
Turkey							+				[58]
United Kingdom	+			+	+		+				[22]
	+				-	-	+		-		[59]
						+					[60]

										-	[61]
United States of America							+				[62]
				+							[63]
				+							[64]
				+							[65]
Vietnam		+	+		+	+	+				[66]

* shellfish were imported from Denmark

+ : virus detected in BMS

- : virus searched but not detected

Empty : virus not searched

References :

1. Mozgovoj, M.; Miño, S.; Barbieri, E.S.; Tort, F.L.; Victoria-Montero, M.; Frydman, C.; Cap, M.; Baron, P.J.; Colina, R.; Matthijnssens, J.; et al. GII.4 Human Norovirus and G8P[1] Bovine-like Rotavirus in Oysters (Crassostrea Gigas) from Argentina. *Int. J. Food Microbiol.* **2022**, *365*, 109553, doi:10.1016/j.ijfoodmicro.2022.109553.
2. Torok, V.; Hodgson, K.; McLeod, C.; Tan, J.; Malhi, N.; Turnbull, A. National Survey of Foodborne Viruses in Australian Oysters at Production. *Food Microbiol.* **2018**, *69*, 196–203, doi:10.1016/j.fm.2017.08.014.
3. Brake, F.; Ross, T.; Holds, G.; Kiermeier, A.; McLeod, C. A Survey of Australian Oysters for the Presence of Human Noroviruses. *Food Microbiol.* **2014**, *44*, 264–270, doi:10.1016/j.fm.2014.06.012.
4. Vasquez-García, A.; Mejia-Ballesteros, J.E.; de Godoy, S.H.S.; Barbieri, E.; de Sousa, R.L.M.; Fernandes, A.M. Norovirus GII and Astrovirus in Shellfish from a Mangrove Region in Cananéia, Brazil: Molecular Detection and Characterization. *Braz. J. Microbiol.* **2021**, *53*, 317–326, doi:10.1007/s42770-021-00631-y.
5. Keller, R.; Justino, J.F.; Cassini, S.T. Assessment of Water and Seafood Microbiology Quality in a Mangrove Region in Vitória, Brazil. *J. Water Health* **2013**, *11*, 573–580, doi:10.2166/wh.2013.245.
6. Souza, D.S.M.; Dominot, A.F.Á.; Moresco, V.; Barardi, C.R.M. Presence of Enteric Viruses, Bioaccumulation and Stability in Anomalocardia Brasiliana Clams (Gmelin, 1791). *Int. J. Food Microbiol.* **2018**, *266*, 363–371, doi:10.1016/j.ijfoodmicro.2017.08.004.
7. Keller, R.; Pratte-Santos, R.; Scarpati, K.; Martins, S.A.; Loss, S.M.; Fumian, T.M.; Miagostovich, M.P.; Cassini, S.T. Surveillance of Enteric Viruses and Thermotolerant Coliforms in Surface Water and Bivalves from a Mangrove Estuary in Southeastern Brazil. *Food Environ. Virol.* **2019**, *11*, 288–296, doi:10.1007/s12560-019-09391-3.
8. do Nascimento, L.G.; Sarmento, S.K.; Leonardo, R.; Gutierrez, M.B.; Malta, F.C.; de Oliveira, J.M.; Guerra, C.R.; Coutinho, R.; Miagostovich, M.P.; Fumian, T.M. Detection and Molecular Characterization of Enteric Viruses in Bivalve Mollusks Collected in Arraial Do Cabo, Rio de Janeiro, Brazil. *Viruses* **2022**, *14*, 2359, doi:10.3390/v14112359.
9. Krumova-Valcheva, G.; Mladenova, Z.; Gogov, Y. Study on Norovirus Contamination of Live Bivalve Molluscs Using Real-Time PCR. *Bulg. J. Vet. Med.* **2020**, *23*, 478–486, doi:10.15547/bjvm.2019-0008.

10. Bonny, P.; Desdouits, M.; Schaeffer, J.; Garry, P.; Essia Ngang, J.J.; Le Guyader, F.S. Contamination of Clams with Human Norovirus and a Novel Hepatitis A Virus in Cameroon. *Food Environ. Virol.* **2020**, *12*, 274–277, doi:10.1007/s12560-020-09432-2.
11. Green, T.J.; Yin Walker, C.; Leduc, S.; Michalchuk, T.; McAllister, J.; Roth, M.; Janes, J.K.; Krogh, E.T. Spatial and Temporal Pattern of Norovirus Dispersal in an Oyster Growing Region in the Northeast Pacific. *Viruses* **2022**, *14*, 762, doi:10.3390/v14040762.
12. Kou, X.; Wu, Q.; Wang, D.; Zhang, J. Simultaneous Detection of Norovirus and Rotavirus in Oysters by Multiplex RT–PCR. *Food Control* **2008**, *19*, 722–726, doi:10.1016/j.foodcont.2007.07.001.
13. Gao, S.; Li, D.; Zha, E.; Zhou, T.; Wang, S.; Yue, X. Surveillance of Hepatitis E Virus Contamination in Shellfish in China. *Int. J. Environ. Res. Public. Health* **2015**, *12*, 2026–2036, doi:10.3390/ijerph120202026.
14. Brnić, D.; Lojkić, I.; Škoko, I.; Krešić, N.; Šimić, I.; Keros, T.; Ganjto, M.; Štefanac, D.; Viduka, B.; Karšaj, D.; et al. SARS-CoV-2 Circulation in Croatian Wastewaters and the Absence of SARS-CoV-2 in Bivalve Molluscan Shellfish. *Environ. Res.* **2022**, *207*, 112638, doi:10.1016/j.envres.2021.112638.
15. Krog, J.S.; Larsen, L.E.; Schultz, A.C. Enteric Porcine Viruses in Farmed Shellfish in Denmark. *Int. J. Food Microbiol.* **2014**, *186*, 105–109, doi:10.1016/j.ijfoodmicro.2014.06.012.
16. Diez-Valcarce, M.; Kokkinos, P.; Söderberg, K.; Bouwknegt, M.; Willems, K.; de Roda-Husman, A.M.; von Bonsdorff, C.-H.; Bellou, M.; Hernández, M.; Maunula, L.; et al. Occurrence of Human Enteric Viruses in Commercial Mussels at Retail Level in Three European Countries. *Food Environ. Virol.* **2012**, *4*, 73–80, doi:10.1007/s12560-012-9078-9.
17. Le Guyader, F.S.; Le Saux, J.-C.; Ambert-Balay, K.; Krol, J.; Serais, O.; Parnaudeau, S.; Giraudon, H.; Delmas, G.; Pommepuy, M.; Pothier, P.; et al. Aichi Virus, Norovirus, Astrovirus, Enterovirus, and Rotavirus Involved in Clinical Cases from a French Oyster-Related Gastroenteritis Outbreak. *J. Clin. Microbiol.* **2008**, *46*, 4011–4017, doi:10.1128/JCM.01044-08.
18. Le Guyader, F.; Haugarreau, L.; Miossec, L.; Dubois, E.; Pommepuy, M. Three-Year Study to Assess Human Enteric Viruses in Shellfish. *Appl. Environ. Microbiol.* **2000**, *66*, 3241–3248, doi:10.1128/AEM.66.8.3241-3248.2000.
19. Grodzki, M.; Ollivier, J.; Le Saux, J.C.; Piquet, J.C.; Noyer, M.; Le Guyader, F.S. Impact of Xynthia Tempest on Viral Contamination of Shellfish. *Appl. Environ. Microbiol.* **2012**, *78*, 3508–3511, doi:10.1128/AEM.07604-11.
20. Grodzki, M.; Schaeffer, J.; Piquet, J.-C.; Le Saux, J.-C.; Chevé, J.; Ollivier, J.; Le Pendu, J.; Le Guyader, F.S. Bioaccumulation Efficiency, Tissue Distribution, and Environmental Occurrence of Hepatitis E Virus in Bivalve Shellfish from France. *Appl. Environ. Microbiol.* **2014**, *80*, 4269–4276, doi:10.1128/AEM.00978-14.
21. Desdouits, M.; Piquet, J.-C.; Wacrenier, C.; Le Mennec, C.; Parnaudeau, S.; Jousse, S.; Rocq, S.; Bigault, L.; Contrant, M.; Garry, P.; et al. Can Shellfish Be Used to Monitor SARS-CoV-2 in the Coastal Environment? *Sci. Total Environ.* **2021**, *778*, 146270, doi:10.1016/j.scitotenv.2021.146270.
22. Formiga-Cruz, M.; Tofiño-Quesada, G.; Bofill-Mas, S.; Lees, D.N.; Henshilwood, K.; Allard, A.K.; Conden-Hansson, A.-C.; Hernroth, B.E.; Vantarakis, A.; Tsibouxi, A.; et al. Distribution of Human Virus Contamination in Shellfish from Different Growing Areas in Greece, Spain, Sweden, and the United Kingdom. *Appl. Environ. Microbiol.* **2002**, *68*, 5990–5998, doi:10.1128/AEM.68.12.5990-5998.2002.
23. Umesha, K.R.; Bhavani, N.C.; Venugopal, M.N.; Karunasagar, I.; Krohne, G.; Karunasagar, I. Prevalence of Human Pathogenic Enteric Viruses in Bivalve Molluscan Shellfish and Cultured Shrimp in South West Coast of India. *Int. J. Food Microbiol.* **2008**, *122*, 279–286, doi:10.1016/j.ijfoodmicro.2007.12.024.
24. Das, O.; Lekshmi, M.; Kumar, S.; Nayak, B.B. Incidence of Norovirus in Tropical Seafood Harbouring Fecal Indicator Bacteria. *Mar. Pollut. Bull.* **2020**, *150*, 110777, doi:10.1016/j.marpolbul.2019.110777.

25. Rajko-Nenow, P.; Keaveney, S.; Flannery, J.; O'Flaherty, V.; Doré, W. Characterisation of Norovirus Contamination in an Irish Shellfishery Using Real-Time RT-QPCR and Sequencing Analysis. *Int. J. Food Microbiol.* **2012**, *160*, 105–112, doi:10.1016/j.ijfoodmicro.2012.10.001.
26. Fiorito, F.; Di Concilio, D.; Lambiase, S.; Amoroso, M.G.; Langellotti, A.L.; Martello, A.; Esposito, M.; Galiero, G.; Fusco, G. Oyster Crassostrea Gigas, a Good Model for Correlating Viral and Chemical Contamination in the Marine Environment. *Mar. Pollut. Bull.* **2021**, *172*, 112825, doi:10.1016/j.marpolbul.2021.112825.
27. Fusco, G.; Anastasio, A.; Kingsley, D.H.; Amoroso, M.G.; Pepe, T.; Fratamico, P.M.; Cio, B.; Rossi, R.; Rosa, G.L.; Boccia, F. Detection of Hepatitis A Virus and Other Enteric Viruses in Shellfish Collected in the Gulf of Naples , Italy. **2019**, *2016*.
28. Purpari, G.; Macaluso, G.; Di Bella, S.; Gucciardi, F.; Mira, F.; Di Marco, P.; Lastra, A.; Petersen, E.; La Rosa, G.; Guercio, A. Molecular Characterization of Human Enteric Viruses in Food, Water Samples, and Surface Swabs in Sicily. *Int. J. Infect. Dis.* **2019**, *80*, 66–72, doi:10.1016/j.ijid.2018.12.011.
29. Fusco, G.; Di Bartolo, I.; Cioffi, B.; Ianiro, G.; Palermo, P.; Monini, M.; Amoroso, M.G. Prevalence of Foodborne Viruses in Mussels in Southern Italy. *Food Environ. Virol.* **2017**, *9*, 187–194, doi:10.1007/s12560-016-9277-x.
30. La Rosa, G.; Fratini, M.; Spuri Vennarucci, V.; Guercio, A.; Purpari, G.; Muscillo, M. GIV Noroviruses and Other Enteric Viruses in Bivalves: A Preliminary Study. *New Microbiol.* **2012**, *35*, 27–34.
31. Mancusi, A.; Capuano, F.; Girardi, S.; Di Maro, O.; Suffredini, E.; Di Concilio, D.; Vassallo, L.; Cuomo, M.C.; Tafuro, M.; Signorelli, D.; et al. Detection of SARS-CoV-2 RNA in Bivalve Mollusks by Droplet Digital RT-PCR (Dd RT-PCR). *Int. J. Environ. Res. Public. Health* **2022**, *19*, 943, doi:10.3390/ijerph19020943.
32. Hansman, G.S.; Oka, T.; Li, T.-C.; Nishio, O.; Noda, M.; Takeda, N. Detection of Human Enteric Viruses in Japanese Clams. *J. Food Prot.* **2008**, *71*, 1689–1695, doi:10.4315/0362-028x-71.8.1689.
33. Hansman, G.S.; Oka, T.; Katayama, K.; Takeda, N. Human Sapoviruses: Genetic Diversity, Recombination, and Classification. *Rev. Med. Virol.* **2007**, *17*, 133–141, doi:10.1002/rmv.533.
34. Yamazaki, Y.; Thongchankaew-Seo, U.; Yamazaki, W. Very Low Likelihood That Cultivated Oysters Are a Vehicle for SARS-CoV-2: 2021-2022 Seasonal Survey at Supermarkets in Kyoto, Japan. *Heliyon* **2022**, *8*, e10864, doi:10.1016/j.heliyon.2022.e10864.
35. Moon, A.; Hwang, I.-G.; Choi, W.S. Prevalence of Noroviruses in Oysters in Korea. *Food Sci. Biotechnol.* **2011**, *20*, 1151–1154, doi:10.1007/s10068-011-0157-8.
36. Choo, Y.-J.; Kim, S.-J. Detection of Human Adenoviruses and Enteroviruses in Korean Oysters Using Cell Culture, Integrated Cell Culture-PCR, and Direct PCR. *J. Microbiol. Seoul Korea* **2006**, *44*, 162–170.
37. Parada-Fabián, J.C.; Juárez-García, P.; Natividad-Bonifacio, I.; Vázquez-Salinas, C.; Quiñones-Ramírez, E.I. Identification of Enteric Viruses in Foods from Mexico City. *Food Environ. Virol.* **2016**, *8*, 215–220, doi:10.1007/s12560-016-9244-6.
38. Ilic, N.; Velebit, B.; Teodorovic, V.; Djordjevic, V.; Karabasil, N.; Vasilev, D.; Djuric, S.; Adzic, B.; Dimitrijevic, M. Influence of Environmental Conditions on Norovirus Presence in Mussels Harvested in Montenegro. *Food Environ. Virol.* **2017**, *9*, 406–414, doi:10.1007/s12560-017-9298-0.
39. Karamoko, Y.; Ibenyassine, K.; Aitmhand, R.; Idaomar, M.; Ennaji, M.M. Adenovirus Detection in Shellfish and Urban Sewage in Morocco (Casablanca Region) by the Polymerase Chain Reaction. *J. Virol. Methods* **2005**, *126*, 135–137, doi:10.1016/j.jviromet.2005.02.003.
40. Benabbes, L.; Ollivier, J.; Schaeffer, J.; Parnaudeau, S.; Rhaissi, H.; Nourlil, J.; Le Guyader, F.S. Norovirus and Other Human Enteric Viruses in Moroccan Shellfish. *Food Environ. Virol.* **2013**, *5*, 35–40, doi:10.1007/s12560-012-9095-8.
41. Boussettine, R.; Hassou, N.; Maanan, M.; Bessi, H.; Ennaji, M.M. Hepatitis A Virus Detection by RT-QPCR in Shellfish Samples from Three Moroccan Atlantic Coastal Areas: Dakhla, Oualidia,

and Moulay Bousselham. *Lett. Appl. Microbiol.* **2023**, *76*, ovac059, doi:10.1093/lambio/ovac059.
42. Lodder-Verschoor, F.; de Roda Husman, A.M.; van den Berg, H.H.J.L.; Stein, A.; van Pelt-Heerschap, H.M.L.; van der Poel, W.H.M. Year-Round Screening of Noncommercial and Commercial Oysters for the Presence of Human Pathogenic Viruses. *J. Food Prot.* **2005**, *68*, 1853–1859, doi:10.4315/0362-028x-68.9.1853.
43. Dirks, R.A.M.; Jansen, C.C.C.; Hägele, G.; Zwartkruis-Nahuis, A.J.T.; Tijsma, A.S.L.; Boxman, I.L.A. Quantitative Levels of Norovirus and Hepatitis A Virus in Bivalve Molluscs Collected along the Food Chain in the Netherlands, 2013–2017. *Int. J. Food Microbiol.* **2021**, *344*, 109089, doi:10.1016/j.ijfoodmicro.2021.109089.
44. Wolf, S.; Hewitt, J.; Greening, G.E. Viral Multiplex Quantitative PCR Assays for Tracking Sources of Fecal Contamination. *Appl. Environ. Microbiol.* **2010**, *76*, 1388–1394, doi:10.1128/AEM.02249-09.
45. Myrmel, M.; Berg, E.M.M.; Rimstad, E.; Grinde, B. Detection of Enteric Viruses in Shellfish from the Norwegian Coast. *Appl. Environ. Microbiol.* **2004**, *70*, 2678–2684, doi:10.1128/AEM.70.5.2678-2684.2004.
46. Tryland, I.; Myrmel, M.; Østensvik, Ø.; Wennberg, A.C.; Robertson, L.J. Impact of Rainfall on the Hygienic Quality of Blue Mussels and Water in Urban Areas in the Inner Oslofjord, Norway. *Mar. Pollut. Bull.* **2014**, *85*, 42–49, doi:10.1016/j.marpolbul.2014.06.028.
47. Bigoraj, E.; Kwit, E.; Chrobocińska, M.; Rzeżutka, A. Occurrence of Norovirus and Hepatitis A Virus in Wild Mussels Collected from the Baltic Sea. *Food Environ. Virol.* **2014**, *6*, 207–212, doi:10.1007/s12560-014-9153-5.
48. Mesquita, J.R.; Vaz, L.; Cerqueira, S.; Castilho, F.; Santos, R.; Monteiro, S.; Manso, C.F.; Romalde, J.L.; Nascimento, M.S.J. Norovirus, Hepatitis A Virus and Enterovirus Presence in Shellfish from High Quality Harvesting Areas in Portugal. *Food Microbiol.* **2011**, *28*, 936–941, doi:10.1016/j.fm.2011.01.005.
49. Rodrigues, I.C.; Santos-Ferreira, N.; Silva, D.; da Silva, C.C.; Inácio, Â.S.; Nascimento, M.S.J.; da Costa, P.M. A One-Year Systematic Study to Assess the Microbiological Profile in Oysters from a Commercial Harvesting Area in Portugal. *Microorganisms* **2023**, *11*, 338, doi:10.3390/microorganisms11020338.
50. Tan, M. t. h.; Ho, S. x.; Chu, J. j. h.; Li, D. Application of Virome Capture Sequencing in Shellfish Sold at Retail Level in Singapore. *Lett. Appl. Microbiol.* **2021**, *73*, 486–494, doi:10.1111/lam.13540.
51. Henigman, U.; Biasizzo, M.; Vadnjal, S.; Toplak, I.; Gombac, M.; Steyer, A.; Prijatelj, M.P.; Ambrožic, M.; Fonda, I.; Kirbiš, A.; et al. Molecular Characterisation of Noroviruses Detected in Mussels (Mytilus Galloprovincialis) from Harvesting Areas in Slovenia.
52. Nagarajan, V.; Chen, J.-S.; Hsu, G.-J.; Chen, H.-P.; Chao, H.-C.; Huang, S.-W.; Tsai, I.-S.; Hsu, B.-M. Surveillance of Adenovirus and Norovirus Contaminants in the Water and Shellfish of Major Oyster Breeding Farms and Fishing Ports in Taiwan. *Pathog. Basel Switz.* **2022**, *11*, 316, doi:10.3390/pathogens11030316.
53. Namsai, A.; Louisirirotchanakul, S.; Wongchinda, N.; Siripanyaphinyo, U.; Virulhakul, P.; Puthavathana, P.; Myint, K.S.; Gannarong, M.; Ittapong, R. Surveillance of Hepatitis A and E Viruses Contamination in Shellfish in Thailand. *Lett. Appl. Microbiol.* **2011**, *53*, 608–613, doi:10.1111/j.1472-765X.2011.03152.x.
54. Kittigul, L.; Panjangampatthana, A.; Rupprom, K.; Pombubpa, K. Genetic Diversity of Rotavirus Strains Circulating in Environmental Water and Bivalve Shellfish in Thailand. **2014**, *3*, 1299–1311, doi:10.3390/ijerph110201299.
55. Kittigul, L.; Thamjaroen, A.; Chiawchan, S.; Chavalitshewinkoon-Petmitr, P.; Pombubpa, K.; Diraphat, P. Prevalence and Molecular Genotyping of Noroviruses in Market Oysters, Mussels, and Cockles in Bangkok, Thailand. *Food Environ. Virol.* **2016**, *8*, 1–8, doi:10.1007/s12560-016-9228-6.

56. Sdiri-Loulizi, K.; Hassine, M.; Aouni, Z.; Gharbi-Khelifi, H.; Chouchane, S.; Sakly, N.; Neji-Guédiche, M.; Pothier, P.; Aouni, M.; Ambert-Balay, K. Detection and Molecular Characterization of Enteric Viruses in Environmental Samples in Monastir, Tunisia between January 2003 and April 2007. *J. Appl. Microbiol.* **2010**, *109*, 1093–1104, doi:10.1111/j.1365-2672.2010.04772.x.
57. Elamri, D.E.; Aouni, M.; Parnaudeau, S.; Le Guyader, F.S. Detection of Human Enteric Viruses in Shellfish Collected in Tunisia. *Lett. Appl. Microbiol.* **2006**, *43*, 399–404, doi:10.1111/j.1472-765X.2006.01978.x.
58. Yilmaz, H.; Bostan, K.; Turan, N.; Muratoglu, K.; Yilmaz, A.; Ozkul, A.A.; Kocazeybek, B.; Helps, C. Real-Time PCR Detection of Norovirus in Mussels Collected from the Bosphorus in Istanbul, Turkey. *Food Environ. Virol.* **2010**, *2*, 64–68, doi:10.1007/s12560-010-9028-3.
59. Farkas, K.; Cooper, D.M.; Mcdonald, J.E.; Malham, S.K.; Rougemont, A.D.; Jones, D.L. Seasonal and Spatial Dynamics of Enteric Viruses in Wastewater and in Riverine and Estuarine Receiving Waters. *Sci. Total Environ.* **2018**, *634*, 1174–1183, doi:10.1016/j.scitotenv.2018.04.038.
60. O'Hara, Z.; Crossan, C.; Craft, J.; Scobie, L. First Report of the Presence of Hepatitis E Virus in Scottish-Harvested Shellfish Purchased at Retail Level. *Food Environ. Virol.* **2018**, *10*, 217–221, doi:10.1007/s12560-018-9337-5.
61. Ransome, E.; Hobbs, F.; Jones, S.; Coleman, C.M.; Harris, N.D.; Woodward, G.; Bell, T.; Trew, J.; Kolarević, S.; Kračun-Kolarević, M.; et al. Evaluating the Transmission Risk of SARS-CoV-2 from Sewage Pollution. *Sci. Total Environ.* **2023**, *858*, 159161, doi:10.1016/j.scitotenv.2022.159161.
62. Costantini, V.; Loisy, F.; Joens, L.; Le Guyader, F.S.; Saif, L.J. Human and Animal Enteric Caliciviruses in Oysters from Different Coastal Regions of the United States. *Appl. Environ. Microbiol.* **2006**, *72*, 1800–1809, doi:10.1128/AEM.72.3.1800-1809.2006.
63. Goyal, S.M.; Gerba, C.P.; Melnick, J.L. Human Enteroviruses in Oysters and Their Overlying Waters. *Appl. Environ. Microbiol.* **1979**, *37*, 572–581, doi:10.1128/aem.37.3.572-581.1979.
64. Connell, C.; Tong, H.-I.; Wang, Z.; Allmann, E.; Lu, Y. New Approaches for Enhanced Detection of Enteroviruses from Hawaiian Environmental Waters. *PloS One* **2012**, *7*, e32442, doi:10.1371/journal.pone.0032442.
65. Ellender, R.D.; Mapp, J.B.; Middlebrooks, B.L.; Cook, D.W.; Cake, E.W. Natural Enterovirus and Fecal Coliform Contamination of Gulf Coast Oysters. *J. Food Prot.* **1980**, *43*, 105–110, doi:10.4315/0362-028X-43.2.105.
66. Suffredini, E.; Le, Q.H.; Di Pasquale, S.; Pham, T.D.; Vicenza, T.; Losardo, M.; To, K.A.; De Medici, D. Occurrence and Molecular Characterization of Enteric Viruses in Bivalve Shellfish Marketed in Vietnam. *Food Control* **2020**, *108*, 106828, doi:10.1016/j.foodcont.2019.106828.
